# Raptor levels are critical for β-cell adaptation to a high-fat diet in male mice

**DOI:** 10.1016/j.molmet.2023.101769

**Published:** 2023-07-07

**Authors:** Manuel Blandino-Rosano, Ruy Andrade Louzada, Joao Pedro Werneck-De-Castro, Camila Lubaczeuski, Joana Almaça, Markus A. Rüegg, Michael N. Hall, Gil Leibowitz, Ernesto Bernal-Mizrachi

**Affiliations:** 1 Department of Internal Medicine, Division of Endocrinology, Diabetes and Metabolism, Miller School of Medicine, University of Miami, Miami, FL, USA; 2 Miami VA Health Care System, Miami, FL, USA; 3 Biozentrum, University of Basel, CH-4056, Basel, Switzerland; 4 Diabetes Unit and Endocrine Service, Hadassah-Hebrew University Medical Center, Jerusalem, Israel

**Keywords:** Islet, Beta-cell, Raptor, High-fat diet, PDX1, FOXA2

## Abstract

**Objective:**

The essential role of raptor/mTORC1 signaling in β-cell survival and insulin processing has been recently demonstrated using raptor knock-out models. Our aim was to evaluate the role of mTORC1 function in adaptation of β-cells to insulin resistant state.

**Method:**

Here, we use mice with heterozygous deletion of *raptor* in β-cells (*βra*^*Het*^) to assess whether reduced mTORC1 function is critical for β-cell function in normal conditions or during β-cell adaptation to high-fat diet (HFD).

**Results:**

Deletion of a raptor allele in β-cells showed no differences at the metabolic level, islets morphology, or β-cell function in mice fed regular chow. Surprisingly, deletion of only one allele of raptor increases apoptosis without altering proliferation rate and is sufficient to impair insulin secretion when fed a HFD. This is accompanied by reduced levels of critical β-cell genes like *Ins1, MafA*, *Ucn3*, *Glut2*, *Glp1r,* and specially PDX1 suggesting an improper β-cell adaptation to HFD.

**Conclusion:**

This study identifies that raptor levels play a key role in maintaining PDX1 levels and β-cell function during the adaptation of β-cell to HFD. Finally, we identified that Raptor levels regulate PDX1 levels and β-cell function during β-cell adaptation to HFD by reduction of the mTORC1-mediated negative feedback and activation of the AKT/FOXA2/PDX1 axis. We suggest that Raptor levels are critical to maintaining PDX1 levels and β-cell function in conditions of insulin resistance in male mice.

## Highlights

• The mTOR signaling pathway is deregulated in human diseases such as cancer and diabetes.
• The role of partial mTORC1 reduction in β-cells during β-cell adaptation to insulin resistance remains unanswered.
• Heterozygous raptor deletion in β-cells impaired HFD adaptation by reducing critical β-cell gene expression.
• Raptor levels are critical for maintenance of PDX1 levels and β-cell function.
• AKT/FOXA2/PDX1 axis plays a role in β-cell adaptation to HFD.

## 1 Introduction

Type 2 diabetes (T2D) is characterized by defective insulin secretion and β-cell expansion in conditions of insulin resistance. β-Cell adaptation to insulin resistance is regulated by the interplay of signals from growth factors, cytokines, insulin, and nutrients such as glucose, fatty acids, and amino acids (AAs). The mTOR complex 1 (mTORC1) is activated by nutrients (AAs, glucose, lipids) and growth factors, thereby linking growth factor, diet, and nutrient excess to β-cell responses. Abnormalities in mTORC1 signaling have been implicated in human diseases including diabetes. Published observations underscore the importance of mTORC1 signaling on modulation of β-cell mass, insulin secretion and adaptation to insulin resistance.

mTORC1 is a protein complex that functions as a nutrient sensor and it is composed of mTOR itself, the regulatory-associated protein of mTOR (Raptor), mammalian lethal with mLST8, PRAS40, and DEPTOR [1]. mTORC1 controls growth, proliferation and metabolism by directly modulating 4 E-BPs and S6 kinases (S6K). One consequence of chronic mTORC1 hyperactivation is the induction of an S6K1-dependent negative feedback loop leading to attenuation of AKT signaling in multiple tissues and insulin resistance [[2], [3], [4], [5]]. Activation of mTORC1 by conditional deletion of TSC2 in β-cells (*βTSC2*^*−/−*^) induces improved glucose tolerance as a result of increased β-cell mass, proliferation and cell size [6]. The importance of endogenous mTORC1 signaling in β-cells has been recently demonstrated using raptor knock-out models [[7], [8], [9]]. These studies have shown a key role of Raptor in proliferation, size, survival, and maturation of the β-cell, and both in function and insulin processing. *In vivo* and *in vitro* studies have shown that inhibition of mTORC1 has a protective effect in conditions of excessive proinsulin misfolding by stimulating autophagy and alleviating ER stress [10,11]. On the other hand, previous studies have suggested the importance of mTORC1 in the adaptation to states of insulin resistance [[10], [11], [12], [13], [14]] including in T2D patients [15] and increased mTORC1 activity has been reported in prediabetic *db/db* mice compared to nondiabetic littermates [14]. Leibowitz et al. demonstrated that blocking mTORC1 by rapamycin in *P. obesus* caused severe impairment of β-cells function, increased β-cells apoptosis, and progression of diabetes [11]. These studies are consistent with other studies showing that the use of rapamycin has been also linked with antiproliferative effects, and alteration in the cell cycle what could negatively impact the adaptation of β-cells to insulin resistance [12]. However, several questions remain unanswered as whether reduced in endogenous mTORC1 function is critical for β-cell function in normal conditions or during β-cell adaptation to insulin resistance. Finally, the role of mTORC1 function in adaptation of β-cells to insulin resistant states has not been directly evaluated.

In the present study, we use mice with heterozygous deletion of *Raptor* in β-cells (*βra*^*Het*^) to assess whether reduced mTORC1 function is critical for β-cell function in normal conditions or during β-cell adaptation to high-fat diet (HFD). *βra*^*Het*^ mice showed no differences at the metabolic level, islets morphology, or β-cell function when fed regular chow. Surprisingly, deletion of only one allele of *Raptor* is sufficient to impair insulin secretion when fed a HFD, increases apoptosis without altering proliferation rate, accompanied by a reduced levels of critical β-cell genes like *Ins1 and 2*, *MafA*, *Ucn3*, *Glut2, Glp1r*, and especially *Pdx1*, suggesting an improper β-cell adaptation to HFD. Our data demonstrate that Raptor protein levels are also of main importance in the maintenance of the PDX1 levels and β-cell function in the adaptation of β-cell to HFD. Finally, our data showing that increasing FOXA2 levels in islets from *βra*^*Het*^ mice exposed to HFD rescue PDX1 levels and insulin secretion highlights the importance of this pathway in HFD adaptation. These set of studies underscore a key role of Raptor levels in maintaining PDX1 levels and β-cell function in conditions of insulin resistance by controlling the mTORC1-dependent negative feedback loop.

## 2 Materials and methods

### 2.1 Animal generation

*RIP-Cre* and *raptor*^*fl/fl*^ mice have been previously described [8,16,17]. Mice with transgenic overexpression of a rapamycin resistant constitutively active form of S6K in β-cells (*caS6K*) and *βraKO;caS6K* have been previously described [8,18]. Studies were performed on mice on C57BL6J background. Results of the experiments are shown for male mice at ages shown in figure legends. All animals were maintained on a 12 h light–dark cycle. All procedures were performed in accordance with the University of Miami-approved protocols.

### 2.2 Metabolic studies

Adult mice were given a normal chow diet or a high-fat diet of 60% kcal fat (D12492, Research Diets). Blood glucose levels were determined from blood obtained from the tail vein using Contour glucometer (Bayer). Fasting glucose and insulin were measured after overnight fasting. Glucose tolerance tests and GSIS were performed on overnight-fasted animals by injecting glucose intraperitoneally (2 and 3 mg kg^−1^, respectively). Plasma insulin and proinsulin levels were determined using a Mouse Ultrasensitive Insulin ELISA kit and Mouse Proinsulin ELISA kit, respectively (ALPCO Immunoassays).

### 2.3 Immunofluorescence staining and morphometric analysis

Formalin-fixed pancreatic tissues were embedded in paraffin. Immunofluorescence staining was performed using primary antibodies described on Supplementary Table 1. Fluorescent images were acquired using a microscope (Leica DM5500B) with a motorized stage using a camera (Leica Microsystems, DFC360FX), interfaced with the OASIS-blue PCI controller, and controlled by the Leica Application Suite X (LAS X). β-Cell ratio assessment was calculated by measuring insulin and acinar areas using Adobe Photoshop 2021 in five insulin-stained sections (5 μm) that were 200 μm apart. To calculate β-cell mass, the β-cell to acinar ratio was then multiplied by the pancreas weight. Islets number was examined using standard histological methods on stained pancreas sections with an insulin antibody. Assessment of proliferation was performed in insulin- and Ki67-stained sections. Apoptosis was determined using TUNEL assay (ApopTag Red in Situ Apoptosis Detection Kit, Chemicon) in insulin-stained sections. At least 3,000 β-cells were counted for each animal. For dispersed cell staining, islets were gently dispersed after 5 min incubation with trypsin–EDTA (0.25% trypsin and 1 mM EDTA) in Hanks’ balanced salt solution without Ca^2+^ and Mg^2+^ (Gibco Invitrogen) at 37 °C followed by fixation in 4% methanol-free formaldehyde onto poly-l-lysine-coated slides. All the morphologic measurements were performed in blinded manner.

### 2.4 Islets studies

After islet isolation, islets were maintained at 37 °C in an atmosphere containing 20% oxygen and 5% CO_2_. Insulin secretion from isolated islets was assessed by static incubation. Briefly, after overnight culture in RPMI containing 5 mM glucose and 10% FBS, islets were pre-cultured for 1 h in Krebs–Ringer medium containing 2 mM glucose. Groups of 10 islets in triplicates were then incubated in Krebs–Ringer medium containing 2 or 16 mM glucose for 1 h. Secreted insulin in the supernatant and insulin content was then measured using Mouse Ultrasensitive Insulin ELISA kit (ALPCO Immunoassays) and normalized to DNA content. Isolated islets were treated *in vitro* with proinflammatory cytokines (IL-1β (50U/ml), IFN-γ and TNF-α (1000U/ml) (Peprotech, Thermo Fisher Scientific), thapsigargin (1 μM) or palmitate (0.4 mM) (Millipore, Bedford, MA). After 24 h treatment, islets were dispersed into a single cell suspension and fixed for flow cytometry analysis.

### 2.5 Western blotting

Islets from an individual mouse (120–150 islets) were lysed in lysis buffer (125 mM Tris, pH 7, 2% SDS and 1 mM dithiothreitol) containing a protease inhibitor cocktail (Roche Diagnostics). Protein quantity was measured by a bicinchoninic acid assay method, and 40 μg of protein were loaded on SDS–PAGE gels and separated by electrophoresis. Separated proteins were transferred onto polyvinylidene difluoride membranes (Millipore, Bedford, MA) overnight. After blocking for 1 h in Li-Cor Blocking buffer, membranes were incubated overnight at 4 °C with a primary antibody diluted in 1 × Tris-buffered saline–1% Tween 20–5% milk followed by 1 h incubation at room temperature with horseradish peroxidase-conjugated secondary antibodies. Antibodies used for immunoblotting are included in Supplementary Table 1, and membranes were developed using Western Bright Sirius Kit (BioExpress). Band densitometry was determined by measuring pixel intensity using NIH Image J software (v1.49 d [19] freely available at http://rsb.info.nih.gov/ij/index.html) and normalized to tubulin, actin or total protein in the same membrane. Images have been cropped for presentation. Full-size images for the most important western blots are presented in Supplementary Figures.

### 2.6 Flow cytometry

After overnight culture in RPMI containing 5 mM glucose, islets were dispersed into a single-cell suspension and fixed with BD Pharmingen Transcription Factor Phospho Buffer Set (BD Biosciences). Dispersed cells were incubated overnight with conjugated antibodies at 4 °C. Dead cells were excluded by Ghost Dye Red 780 (Tonbo), and signal intensity from single stained cells and GFP was analyzed by mean fluorescent intensity in insulin-positive cells using BD LSR II (BD Biosciences). Antibodies used are included in Supplementary Table 1.

### 2.7 Quantitative real-time PCR

Total RNA was isolated using RNeasy (Qiagen) followed by cDNA synthesis using High-Capacity cDNA Reverse Transcription Kit (Applied Biosystems) according to the manufacturer's protocol. Real-time PCR was performed on an ABI 7000 sequence detection system using POWER SYBR-Green PCR Master MIX (Applied Biosystems). Primers were purchased from IDT Technologies. Primer pair for:

*pdx1* was as follows: 5′-CCC CAG TTT ACA AGC TCG CT-3′ (forward); 5′-CTC GGT TCC ATT CGG GAA AGG-3′ (reverse), *ins1*: 5′-CAC CCC ACC TGG AGA CCT TA-3′ (forward); 5′-TGA AAC AAT GAC CTG CTT GCT G-3′ (reverse) and *ins2*: 5′-GCA AGC AGG AAG GTT ATT GTT TCA-3′ (forward); 5′-GCT TGA CAA AAG CCT GGG TG-3′ (reverse).

### 2.8 RNA-Seq library preparation, sequencing, and data analysis

RNA Quality Control and DNase Treatment: A total of 12 mice islets RNA samples were submitted to Ocean Ridge Biosciences (Deerfield Beach, FL) for mRNA-Sequencing. Total RNA was quantified by O.D. measurement and assessed for quality on a 1% agarose – 2% formaldehyde RNA Quality Control (QC) gel. The RNA was then digested with RNase free DNase I (Epicentre; Part #D9905K) and re-purified using Agencourt RNAClean XP beads (Beckman Coulter; Part # A63987). The newly digested RNA samples were then quantified by O.D. measurement. The newly digested RNA samples were then quantified by O.D. measurement and checked for quality.

Library Preparation: Amplified cDNA libraries suitable for sequencing were prepared from 250 ng (ng) of DNA-free total RNA using the TruSeq Stranded mRNA Library Prep (Illumina Inc.; Part # 20,020,595). The quality and size distribution of the amplified libraries were determined by chip-based capillary electrophoresis (Bioanalyzer 2100, Agilent Technologies). Libraries were quantified using the KAPA Library Quantification Kit (Kapa Biosystems, Boston, MA).

### 2.9 Sequencing

The 8 libraries were pooled at equimolar concentrations and sequenced in a total of 3 runs on the Illumina NextSeq 500 sequencer using two Mid Output v2 150 cycle kits (part# FC-404-2001) and one High Output v2.5 150 cycle kit (part# 20,024,907). In each case the libraries were sequenced with 76 nt paired-end reads plus 8 nt dual-index reads on the instrument running NextSeq Control Software version 2.2.0.4. Real time image analysis and base calling were performed on the instrument using the Real-Time Analysis (RTA) software version 2.4.11. Generation of FASTQ files: Base calls from the NextSeq 500 RTA were converted to sequencing reads in FASTQ format using Illumina's bcl2fastq program v2.17.1.14 with default settings. Sequencing adapters were not trimmed in this step.

### 2.10 Adenoviral infection

After overnight culture in RPMI containing 5 mM glucose, islets were infected with adenoviruses carrying the cytomegalovirus promoter (Ad. CMV) or FOXA2 and GFP under the control of the CMV promoter (Ad. FOXA2-GFP (ADV-209226), Vector BioLabs). The particle:plaque-forming unit ratio of the stock virus used in the experiments was 300.

### 2.11 Statistical analysis

Data are presented as mean ± s.e.m. and were considered statistically significant when the *P* value was <0.05. Student's unpaired *t* test was used to assess statistical difference between 2 groups using Prism version 9 (GraphPad Software, San Diego, CA). Comparison between more than 2 groups was performed using 2-way ANOVA with repeated measures followed by post hoc 2-tailed Student's *t* tests. The results were considered statistically significant when the *p* value was equal than 0.05.

### 2.12 Data availability

All relevant data are available from the authors on request.

## 3 Results

### 3.1 Heterozygous raptor deletion in β-cells exhibits normal glucose homeostasis

**Figure 1:**
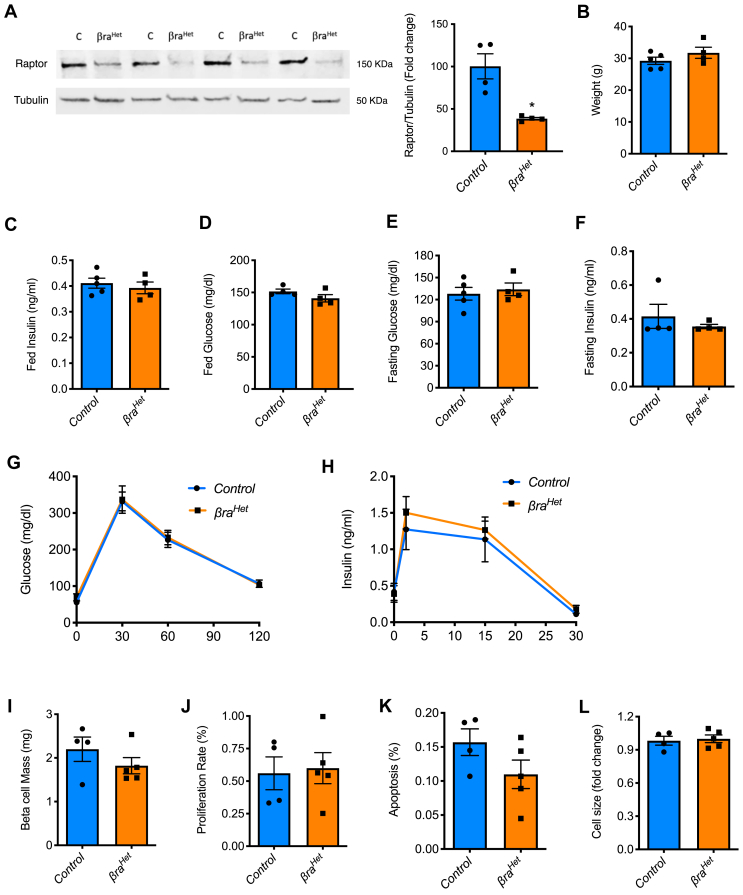
***βra***^***Het***^***exhibits normal glucose homeostasis.*** (A) Immunoblotting and quantification of raptor and tubulin in *control* and *βra*^*Het*^ islets. (B–F) Body weight, random fed serum insulin levels, random fed glucose levels, fasting glucose levels and fasting insulin levels in *control* and *βra*^*Het*^ mice at 3 months of age. (G) Intraperitoneal glucose tolerance test (IPGTT) at 3 months of age. (H) Glucos estimulated insulin secretion (GSIS) in 3-month-old mice. (I–L) Assessments of β-cell mass, proliferation rate by Ki67 staining, apoptosis by TUNEL, and cell size in β-cells. (n ≥ 4). Data expressed as means ± s.e.m.

To decrease endogenous mTORC1 function, we generated mice with heterozygous deletion of *Raptor* in β-cells by crossing *raptor*^*f/f*^ with *Rip-Cre* mice (*βra*^*Het*^) (Figure 1A), both previously described [8,16,17]. To further test if decreased Raptor levels would affect mTORC1 activity, we performed starvation/refeeding with AAs to assess the phosphorylation of S6. Islets from *βra*^*Het*^ exhibit decreased phosphorylation of S6 after stimulation with amino acids, indicating that β-cells from *βra*^*Het*^ are not able to fully activate mTORC1 (Supp Figure. 1a). However, weight, random fed and fasting blood glucose and insulin levels were normal in the *βra*^*Het*^ mice at 3 months of age (Figure 1B–F). Examination of glucose tolerance and glucose-stimulated insulin secretion in *βra*^*Het*^ mice showed no differences when compared to the control mice (*raptor*^*f/f*^ *and Rip-Cre*) at 3 months of age (Figure 1G–H). These studies suggest that, in contrast to *βraKO* mice (homozygous deletion of *raptor* in β-cells) [8], deletion of one raptor allele (*βra*^*Het*^) displayed normal glucose levels and glucose tolerance in regular chow (RC). Consistent with the results in glucose homeostasis, *βra*^*Het*^ mice exhibited normal β-cell mass with similar levels in proliferation, survival, and cell size (Figure 1I-L). While we previously demonstrated that Raptor/mTORC1 is necessary for maintaining postnatal β-cell mass by controlling apoptosis, size, and proliferation [8], the heterozygous deletion of *Raptor* does not appear to affect the maintenance of postnatal β-cell mass.

### 3.2 Heterozygous raptor deletion in β-cells exhibits normal insulin secretion and intracellular calcium responses *ex vivo*

**Figure 2:**
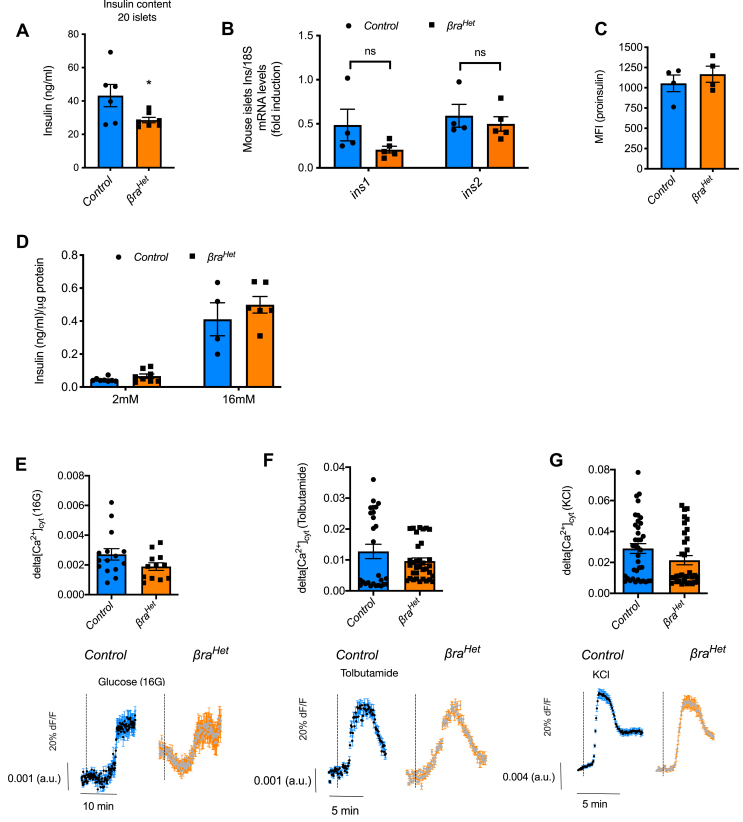
***βra***^***Het***^***exhibits normal insulin secretion.*** (A) Assessment of insulin content in 20 islets equivalent by ELISA from *control* and *βra*^*Het*^ mice at 3 months of age. (B) Ins1 and Ins2 expression at the mRNA level by RT-PCR in islets from *control* and *βra*^*Het*^ mice. (C) Assessment of proinsulin levels in insulin positive cells by FACS. (D) Glucose stimulated insulin secretion (GSIS) *in vitro* using isolated islets from 3-month-old mice. (n ≥ 4). (E–G) Quantification (left) and traces (right) showing relative changes in [Ca^2+^]_i_ (fluorescence (Fluo-4M)) induced by glucose (16 mM), tolbutamide, and KCl in islets from *control* and *βra*^*Het*^ mice. The numbers of cells analyzed are shown as dots. Data are from three different mice. Data expressed as means ± s.e.m., ∗*p* < 0.05.

The results obtained with the *βra*^*Het*^ mice indicate that decreased mTORC1 is not critical for β-cell mass maintenance. While glucose tolerance was normal *in vivo*, assessment of insulin content demonstrated a significant decrease in islets from *βra*^*Het*^ mice (Figure 2A); however, the decrease in insulin content was not due to a decrease in mRNA levels (Figure 2B). Moreover, and in contrast to *βraKO* mice [8], proinsulin levels were not increased in β-cells from *βra*^*Het*^ mice compared to controls (Figure 2C). To further characterize the normal glucose tolerance, we measured β-cell function by static incubation and glucose-mediated calcium imaging in islets from *βra*^*Het*^ and control mice. Glucose-dependent insulin release by static incubation exhibited no differences between islets from *βra*^*Het*^ and control (Figure 2D). To complement the insulin secretory responses, Ca^2+^ imaging using the calcium indicator Fluo-4AM was determined in islets from *βra*^*Het*^ and control mice. Intracellular Ca^2+^ responses to secretagogues such as 16 mM glucose, tolbutamide, or 30 mM KCl were not significantly different between *βra*^*Het*^ and control islets (Figure 2E–G). These studies demonstrate that although β-cells from *βra*^*Het*^ mice exhibit a slight decrease in insulin content, this was not sufficient to alter glucose tolerance at 3 months old (Figure 1C–H) and the responses to glucose and other secretagogues in regular conditions were conserved.

### 3.3 *βra*^*Het*^ mice exhibit impaired glucose homeostasis in HFD

**Figure 3:**
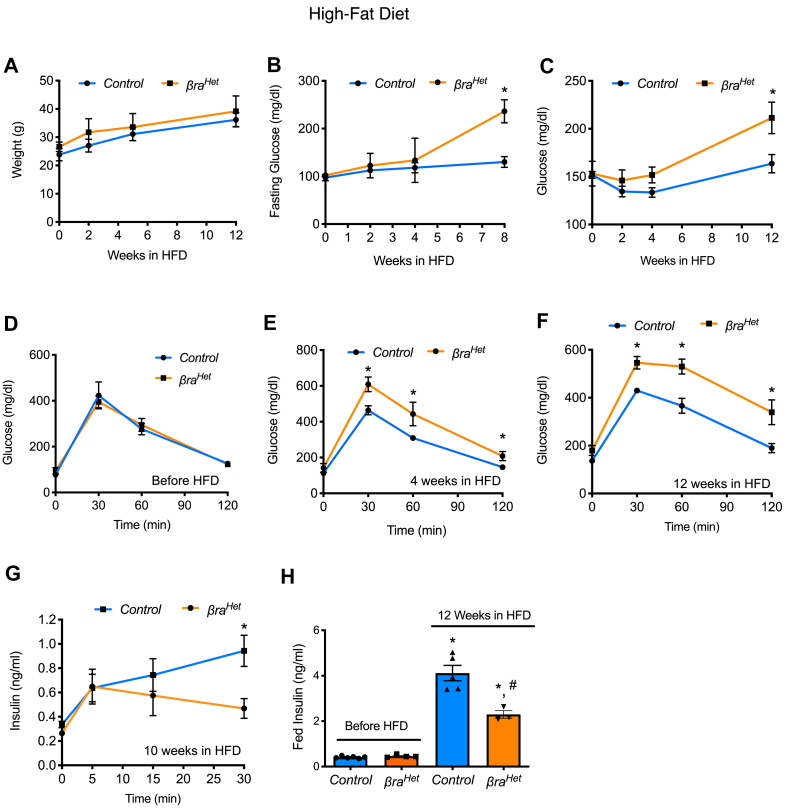
***βra***^***Het***^***mice exhibit impaired glucose homeostasis in HFD.*** (A–C) Body weight, fasting glucose levels and random fed glucose levels in *control* and *βra*^*Het*^ mice during 12 weeks in HFD. (D) IPGTT before HFD at 2 months of age. (E–F) IPGTT after 4 and 12 weeks in HFD. (G) Glucose stimulated insulin secretion (GSIS) in after 10 weeks in HFD. (n ≥ 4) (H) Random fed serum insulin levels before and after 12 weeks in HFD. Data expressed as means ± s.e.m., ∗*p* < 0.05 compared to control before HFD and ^#^*p* < 0.05 compared to *βra*^*Het*^ before HFD.

Given that *βra*^*Het*^ mice fed-RC showed no significant differences in glucose homeostasis, islet morphology and β-cell function, we decided to assess the adaptation to insulin resistance by administering HFD for 12 weeks. In response to HFD, *βra*^*Het*^ and control littermates exhibited similar weight through the 12 weeks experiment (Figure 3A). Interestingly, after 8–12 weeks in HFD, *βra*^*Het*^ mice exhibit higher fasting- and fed/random glucose levels (Figure 3B–C) suggesting that both *Raptor* alleles are necessary for β-cell adaptation to HFD. While glucose tolerance was normal before HFD (Figure 3D), *βra*^*Het*^ mice fed HFD developed abnormal glucose tolerance by 4 weeks, and this was more severely impaired at 12 weeks (Figure 3E–F). Consistent with these results, glucose-stimulated insulin secretion (GSIS) was also impaired in *βra*^*Het*^ mice at 10 weeks of HFD (Figure 3G). In agreement with these results, although random-fed insulin values in *βra*^*Het*^ mice were increased after 12 weeks of HFD, this increase was significantly less than in controls (Figure 3H).

### 3.4 β-cells from heterozygous raptor mice exhibit reduced β-cell survival and area and are more susceptible to palmitate treatment *in vitro*

**Figure 4:**
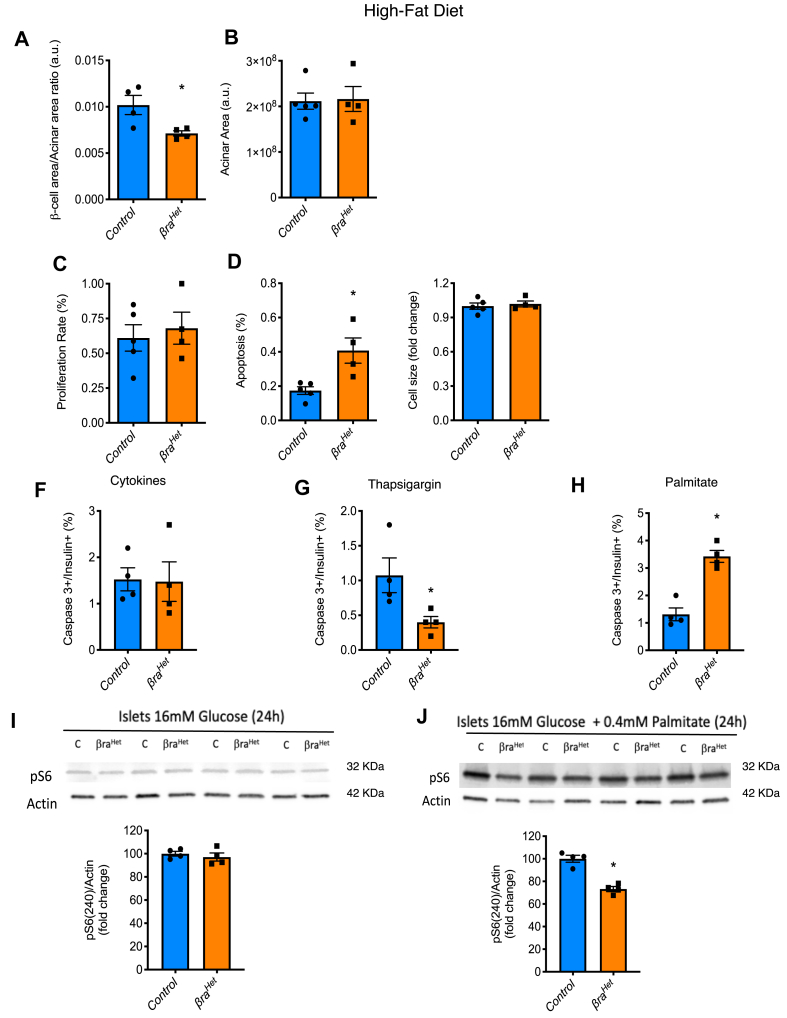
***β-Cells with heterozygous raptor deletion are more susceptible to die by lipotoxicity.*** (A–E) Assessments of β-cell and acinar area, proliferation rate by Ki67 staining, apoptosis by TUNEL, and cell size in β-cells in pancreas sections from *control* and *βra*^*Het*^ mice exposed to HFD for 12 weeks. (F–H) Caspase 3 positive in insulin positive cells by FACS in dispersed cells from islets treated *in vitro* with proinflammatory cytokines (IL-1β (50U/ml), IFN-γ and TNF-α (1000U/ml), thapsigargin (1 μM) or palmitate (0.4 mM) for 24 h. (I–J) Immunoblotting and quantification of pS6 (240) and actin in *control* and *βra*^*Het*^ islets treated with high glucose (16 mM) with or without palmitate (0.4 mM) for 24 h. (n ≥ 4). Data expressed as means ± s.e.m., ∗*p* < 0.05.

Assessment of pancreas morphology showed a decreased in β-cell area and no differences in acinar area (Figure 4A–B), or islets number (*control* 81.33 ± 19.67 *n* = 6 vs *βra*^*Het*^ 101 ± 10.58, *n* = 4, *p* = 0.1) after 12 weeks in HFD. A decreased in β-cell area could be due to decreased size and/or proliferation or increased apoptosis. Assessment of proliferation by Ki67, apoptosis by TUNEL, and size showed that β-cells from *βra*^*Het*^ mice exhibit higher levels of apoptosis when challenged to HFD (Figure 4C–E). We next investigated the mechanisms responsible for β-cell loss in *βra*^*Het*^ mice in HFD by assessing apoptosis in isolated islets exposed for 24 h to ER stress, oxidative stress inducers such as proinflammatory cytokines, and lipotoxicity. Assessment of apoptosis in insulin positive cells by cleaved-caspase 3 levels in dispersed islets using FACs showed no differences between *βra*^*Het*^ and control β-cells after treatment with proinflammatory cytokines (IL1-β (50U/ml), TNF-α and IFN-γ (1000U/ml) (Figure 4F). Unexpectedly, β-cells from *βra*^*Het*^ mice were more resistant to thapsigargin (Figure 4G). In contrast, β-cells from *βra*^*Het*^ mice were more susceptible to apoptosis induced by lipotoxicity (Figure 4H), indicating that β-cells from *βra*^*Het*^ islets are particularly sensitive to conditions of excess of lipids. To further dissect the effect of glucose and palmitate on mTORC1 activation, we designed experiments to assess mTORC1 activity by S6 phosphorylation in high glucose and glucolipotoxicity conditions. No difference in mTORC1 activation measured by S6 phosphorylation was found when the islets were cultured only with high glucose (16 mM) for 24 h (Figure 4I). In contrast, a 30% decreased in S6 phosphorylation was observed when the islets were cultured with high glucose + palmitate (Figure 4J). Together, these data demonstrate that deletion of one allele of *Raptor* has no effect in activation of mTORC1 signaling by high levels of glucose, but it is involved in the response to high glucose and palmitate.

### 3.5 β-cell function is affected in *βra*^*Het*^ mice fed HFD

**Figure 5:**
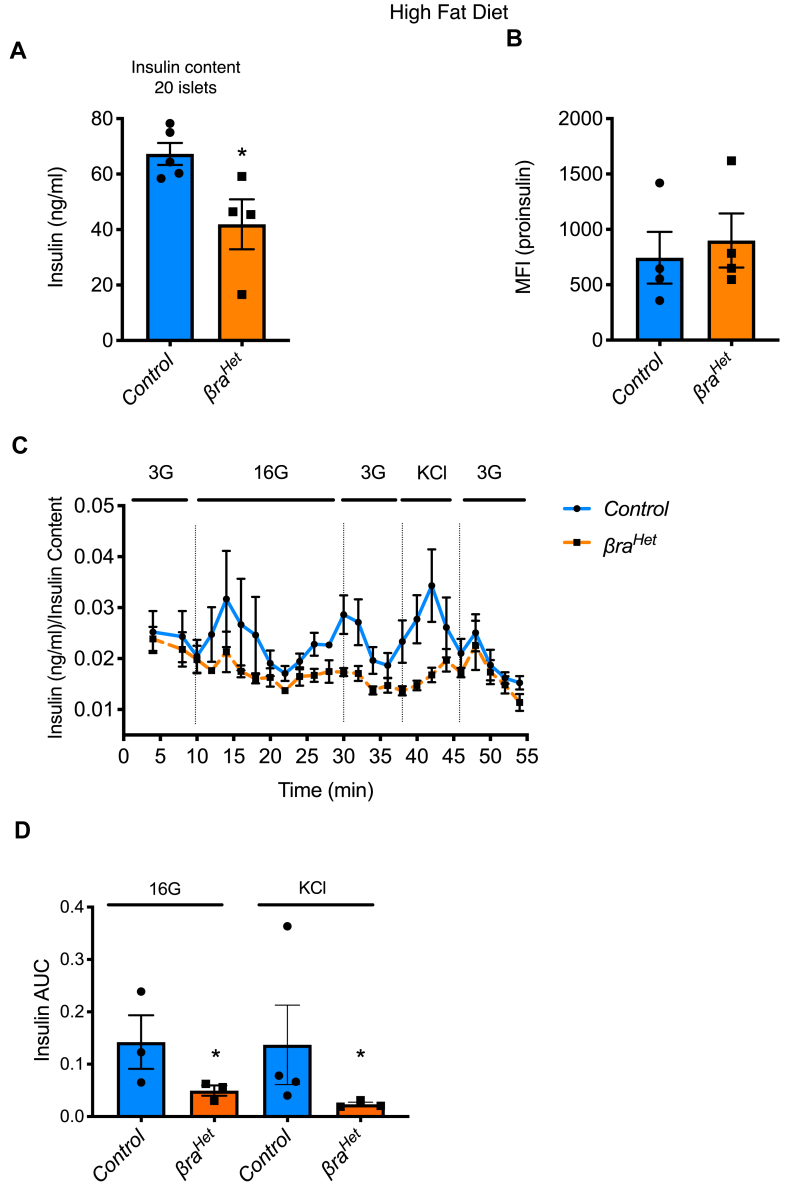
***β-Cell function is impaired in βra***^***Het***^***mice fed HFD.*** (A) Assessment of insulin content in 20 islets equivalent by ELISA. (B) Assessment of proinsulin levels in insulin positive cells by FACS after 12 weeks in HFD. (C–D) Assessment of dynamic glucose-responsive insulin secretion (perifusion) and quantification, in isolated islets from *βra*^*Het*^ mice fed HFD for 12 weeks. (n ≥ 3). Data expressed as means ± s.e.m., ∗*p* < 0.05.

We next designed experiments to assess the mechanisms responsible for the abnormalities in insulin secretion in *βra*^*Het*^ and control mice fed HFD. Similar to the findings in RC (Figure 2A,C), islets from *βra*^*Het*^ mice fed HFD also exhibit a decrease in insulin content (Figure 5A vs 2a) and conserved insulin processing measured by proinsulin levels (Figure 5B vs 2c). Dynamic glucose-responsive insulin secretion by islet perifusion studies using isolated islets from *βra*^*Het*^ mice fed HFD showed a decreased response to 16 mM glucose and KCl when compared to control islets (Figure 5C–D). All together, these data suggest that exposure to HFD uncovered a defect in β-cell function in *βra*^*Het*^ mice.

### 3.6 *βra*^*Het*^ mice fed HFD exhibit decreased levels of β-cell identity markers

**Figure 6:**
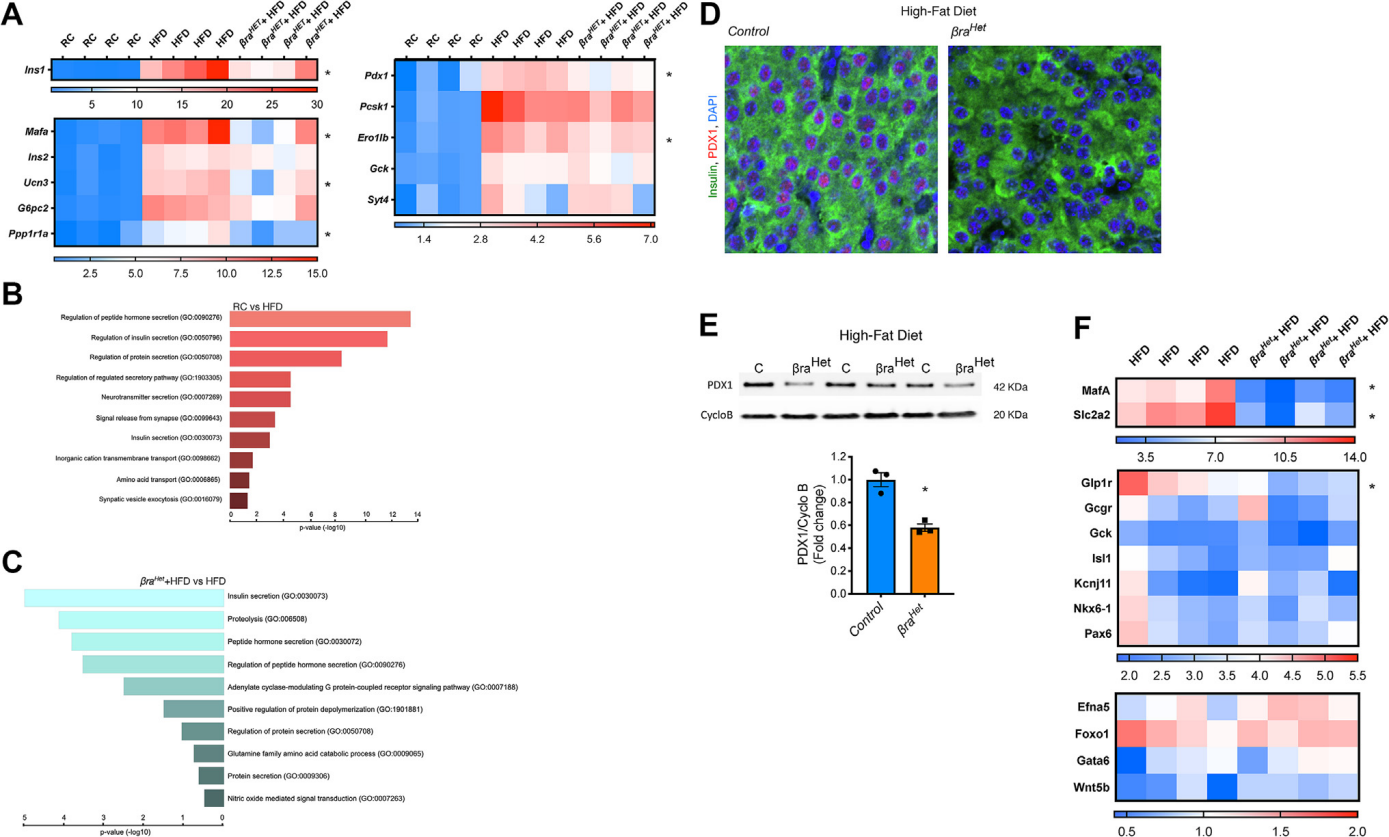
***βra***^***Het***^***mice fed HFD exhibit decreased levels of β-cell markers including PDX1.*** (A) Gene-expression heatmap of the differentially expressed genes related to β-cell maturation in *control* mice fed regular chow (RC) or high-fat diet (HFD) and *βra*^*Het*^ mice fed HFD for 8 weeks. Genes are represented in rows and mice in columns. (B–C) GO-driven pathway analysis of differentially expressed genes with >1.5-fold change between islets fed RC and islets fed HFD for 8 weeks and with <0.5-fold change between *βra*^*Het*^ and *control* islets from mice exposed for 8 weeks to HFD. (D) Immunostaining of insulin (green), PDX1 (red) and DAPI (blue) in pancreas sections from *control* and *βra*^*Het*^ mice exposed to HFD for 12 weeks. Scale bar, 20 μm. (E) Immunoblotting and quantification of PDX1 and Cyclophilin B in *control* and *βra*^*Het*^ islets from mice fed HFD for 12 weeks. (F) Gene-expression heatmap of the differentially expressed genes related to PDX1 downstream targets in *control* and *βra*^*Het*^ mice fed HFD for 8 weeks. Genes are represented in rows and mice in columns. (n ≥ 4). Data expressed as means ± s.e.m., ∗*p* < 0.05.

The previous results indicate that β-cell function and mass in *βra*^*Het*^ islets are reduced after HFD. To obtain a mechanistic insight for these abnormalities, we performed RNAseq in islets from control mice fed regular chow (RC), control mice fed HFD (HFD), and *βra*^*Het*^ fed HFD (*βra*^*Het*^ + HFD) for 8 weeks. Analyzes of tissue-cell-specific genes related and acinar genes and using the meta-analysis tools Metascape [20] and TRRUST [21] to evaluate acinar cell contamination in the RNAseq datasets (Supp Figures. 2a–b) shows no significant differences among the three groups. Next, we determined the effects of heterozygous deletion of Raptor in HFD by comparing the RC, HFD and *βra*^*Het*^ + HFD data sets. The unbiased RNAseq analysis revealed 962 affected genes (RC vs HFD) (Figure 6 and Supp Figure. 2c). This analysis of key β-cell identity genes showed that mRNA for *Ins1*, *MafA*, *Ucn3*, *Ppp1r1a, Pdx1* and *Ero1b* were significantly increased in HFD compared to RC (Figure 6A). The increase in these identity genes by HFD was significantly reduced in *βra*^*Het*^ + HFD (Figure 6A). More importantly, *Pdx1* mRNA was also lower in *βra*^*Het*^ + HFD mice when compared to HFD littermates (Figure 6A). To identify the biological processes induced by HFD and the role of *Raptor* heterozygous deletion, we first performed Gene Ontology (GO) analysis on differentially expressed genes (DEGs) between RC and HFD groups (Figure 6B). This analysis showed that HFD regulates critical genes for β-cell function such as hormone/protein secretion, insulin secretion, secretory pathways, and vesicle exocytosis (Figure 6B). Then, we performed GO analysis on DEGs between HFD and *βra*^*Het*^-HFD mice (Figure 6C). This study showed that β-cells with heterozygous *Raptor* deletion displayed a decreased in insulin/hormone/protein secretions, and proteolysis defects among others (Figure 6C). The abnormalities in insulin secretion and increase in apoptosis are reminiscent of the phenotype observed in PDX1 heterozygous mice [22,23] and led us to hypothesize that PDX1 reduction could be responsible, at least in part, for the *βra*^*Het*^-HFD phenotype. In addition, previous studies by Hagman et al. showed that palmitate inhibits insulin gene expression by reducing the binding of PDX1 and MafA to the insulin promoter [24]. To test this hypothesis, we first assessed PDX1 levels in HFD and *βra*^*Het*^-HFD. A decrease in PDX1 was observed at the protein level by immunostaining and immunoblotting in *βra*^*Het*^-HFD mice for 12 weeks (Figure 6D–E). Evaluation of PDX1 protein levels by immunoblotting showed no differences in islets from *βra*^*Het*^ compared to control littermates in RC (Supp Figure. 3). The reduction in PDX1 levels exclusively in *βra*^*Het*^-HFD could explain in part the differences in β-cell area and function in HFD but not in RC (Figure 1, Figure 2). Analysis of PDX1 target genes in the RNAseq data shows that in addition to *MafA*, other important downstream targets of PDX1 such as *Glp1r*, and *Slc2a2* (Glut2) [25,26] were also decreased in *βra*^*Het*^-HFD mice compared to HFD mice (Figure 6F).

### 3.7 *βra*^*Het*^ mice fed HFD exhibit decreased levels of PDX1 and nuclear FOXA2

**Figure 7:**
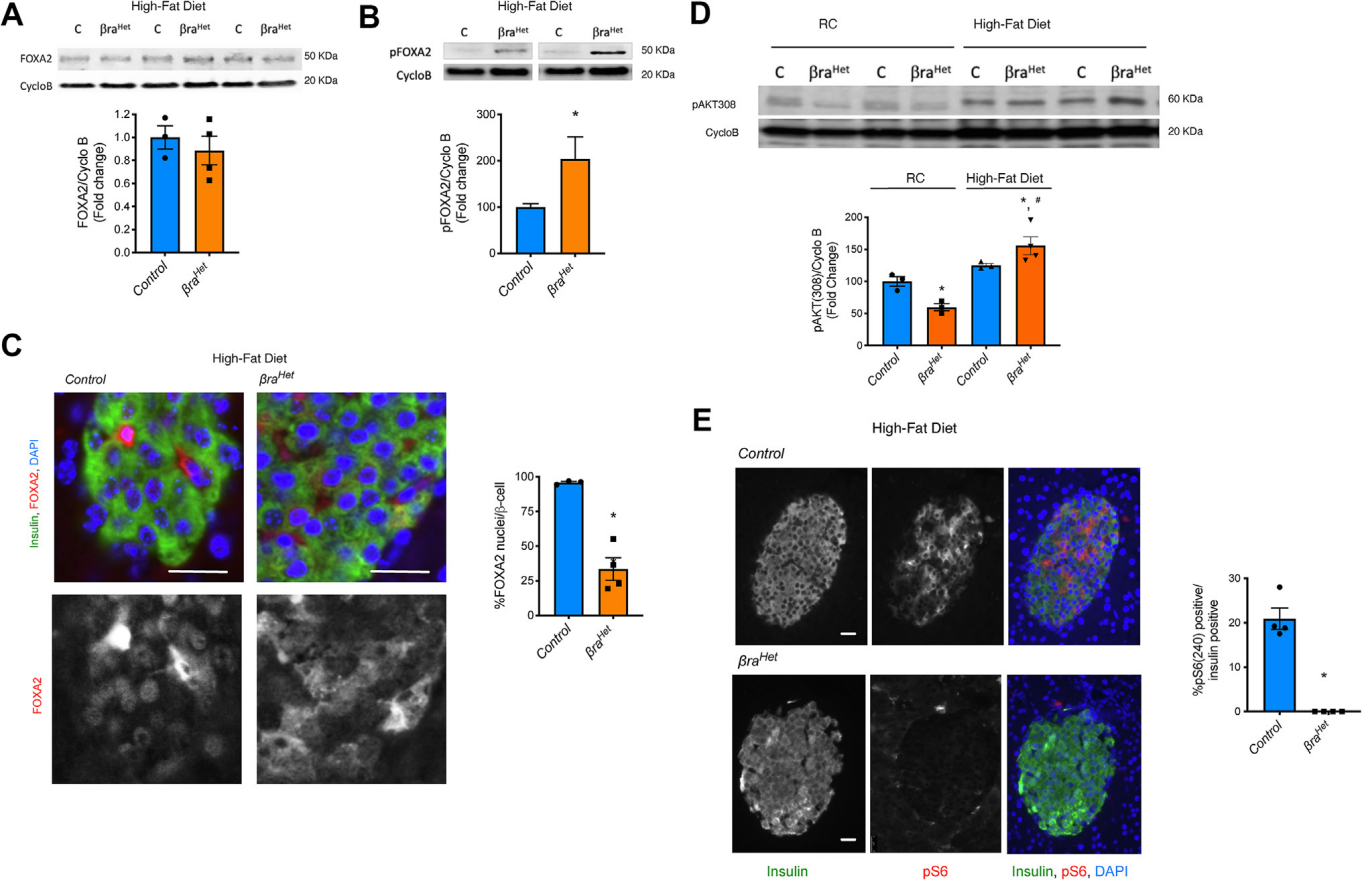
***βra***^***Het***^***mice fed HFD exhibit decreased levels of nuclei FOXA2.*** (A) Immunoblotting and quantification of FOXA2 and Cyclophilin B in *control* and *βra*^*Het*^ islets from mice fed HFD for 12 weeks. (B) Immunoblotting and quantification of pFOXA2 and Cyclophilin B in *control* and *βra*^*Het*^ islets from mice fed HFD for 12 weeks. (C) Quantification and staining of nuclear FOXA2 in β-cells from *control* and *βra*^*Het*^ mice exposed to HFD for 12 weeks. Insulin (green), FOXA2 (red, upper and white, lower) and DAPI (blue). Scale bar, 20 μm. (D) Immunoblotting and quantification of pAKT (Thr308) and Cyclophilin B in *control* and *βra*^*Het*^ islets from mice fed HFD for 12 weeks. (E) Quantification and staining of pS6 (Ser240) in β-cells from *control* and *βra*^*Het*^ mice exposed to HFD for 12 weeks. Insulin (left), pS6 (center) and merge with DAPI (right). (n ≥ 4). Data expressed as means ± s.e.m., ∗*p* < 0.05.

Previous work has shown that PDX1 is regulated by different transcription factors, including HNF-3β/FOXA2, HNF6, HNF-1α, HNF-1β, SP1/3, USF1/2, and PDX-1 itself [27,28]. Analysis of these transcription factors in the RNAseq data showed no differences in these *Pdx1* transcriptional regulators among RC, HFD and *βra*^*Het*^-HFD suggesting that some of these transcription factors could be regulated at the protein level or cellular localization (Supp Figure. 4a). Interestingly, analysis by TRRUST of PDX1 regulators and transcription factors that share targets with PDX1 confirm the synergism of HNF6, MafA, FOXA2 and PDX1 in regulation of β-cell identity (Supp Figures. 4b–d) [25]. FOXA2 has been previously implicated in pancreatic development and β-cell maturation acting upstream of PDX1 [27,29], and its cooperative function with PDX1 is critical for proper β-cell function [25]. In addition, our group recently identified a novel link between mTORC1 and FOXA2 in transcriptional regulation in α-cells [30]. Based on these studies, we hypothesized that FOXA2 and PDX1 could be involved in the adaptation defect observed in *βra*^*Het*^-HFD. FOXA2 expression levels were similar in control and *βra*^*Het*^ islets from mice fed HFD for 12 weeks (Figure 7A). To assess if FOXA2 activation and nuclear/cytosolic localization plays a role in this process we assessed the phosphorylation of FOXA2. Phosphorylation of FOXA2 in T156 was significantly elevated in *βra*^*Het*^-HFD when compared to control-HFD but not in RC (Figure 7B and Supp Figure. 5). This suggests that AKT activity could be differentially induced in *βra*^*Het*^-HFD as T156 phosphorylation of FOXA2 is mediated by AKT [31]. Since AKT has been previously shown to regulate PDX1 transcription by inducing phosphorylation and exclusion of FOXA2 to the cytosol [31], we assessed FOXA2 cellular localization. Immunostaining and quantification of pancreas sections demonstrated a 70% decreased in nuclear FOXA2 staining in β-cells from *βra*^*Het*^ mice fed HFD (Figure 7C). Indeed, AKT (T308) phosphorylation was significantly elevated in *βra*^*Het*^-HFD islets compared to control-HFD and it was increased by almost 3-fold when compared to islets from *βra*^*Het*^ RC-fed mice (Figure 7D). Consistent with reduction in mTORC1 activity, phosphorylation of S6 (Ser240) was totally blunted in *βra*^*Het*^-HFD (Figure 7E). Therefore, we hypothesized that the decreased levels of S6 phosphorylation in response to palmitate (Figure 4J) and S6 phosphorylation in *βra*^*Het*^-HFD was associated with a decreased in the previously described mTORC1-mediated feedback inhibition on IRS1/2 [3,4,32] and AKT phosphorylation, ultimately affecting PDX1 levels.

### 3.8 Overexpression of FOXA2 in *βra*^*Het*^ mice fed HFD rescues PDX1 levels and insulin secretion

**Figure 8:**
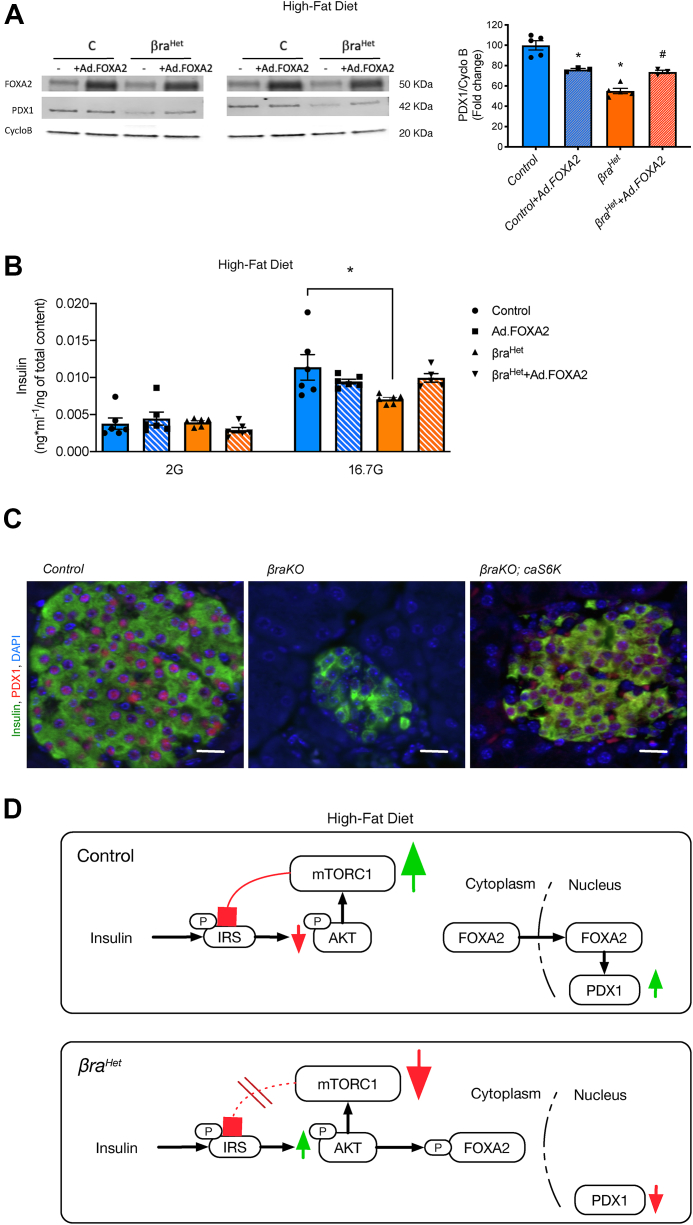
***Overexpressing FOXA2 levels in βra***^***Het***^***mice fed HFD rescue PDX1 levels and insulin secretion.*** (A) Immunoblotting and quantification of FOXA2, PDX1 and Cyclophilin B in islets from *control* and *βra*^*Het*^ mice fed HFD for 12 weeks infected with a control (Ad. CMV, -) or a FOXA2-GFP adenovirus (+Ad. FOXA2). (B) Glucose stimulated insulin secretion (GSIS) *in vitro* using isolated islets in the same conditions. (n ≥ 4). Data expressed as means ± s.e.m., ∗*p* < 0.05. (C) Immunostaining of PDX1 (red), insulin (green) and Dapi (blue) in pancreas sections from *control*, *βraKO,* and *βraKO; caS6K.* Images are representative of three different mice. (D) Schematic summarizes the results of the current experiments by showing how decrease in mTORC1 activity activates AKT/FOXA2 axis and regulates PDX1 during β-cell adaptation to HFD in conditions of reduced mTORC1 activity. In wild type mice conditions, HFD induced insulin resistance activates AKT and mTORC1 signaling. However, sustained activation of mTORC1/S6K axis inhibits insulin/AKT signaling by a negative feedback loop mediated by phosphorylation and degradation of IRS1/2. The decrease in AKT activity reduces phosphorylation and nuclear translocation of its target FOXA2 which in turns induces PDX1 transcription and β-cell maturation. In conditions of decreased mTORC1 activation (*βra*^*Het*^*mice***)**, the decrease in mTORC1 activity results in de-repression of the negative feedback loop and AKT activation. Increase in AKT activity causes a reduction in FOXA2 phosphorylation and cytoplasmic retention leading to decrease in PDX1 levels.

To determine if FOXA2 was responsible for the decreased levels of PDX1 and the function defect of *βra*^*Het*^ islets in HFD, FOXA2 levels were increased by transduction with control or FOXA2-GFP adenoviruses (Ad. FOXA2-GFP^+^) in islets from control and *βra*^*Het*^ mice HFD-fed for 12 weeks. Immunostaining studies were performed to confirm the increased expression of FOXA2 in β-cells nuclei (Supp Figure. 6). As previously shown, PDX1 levels were reduced in islets from *βra*^*Het*^ compared to controls mice (Figure 8A). PDX1 levels are reduced by 20% in islets from control mice fed HFD cultured with the control adenovirus (Figure 8A). Interestingly, an increase in FOXA2 levels is sufficient to rescue PDX1 levels significantly in *βra*^*Het*^ islets similar to the control + adenovirus level (Figure 8A). Moreover, to test whether these increases in FOXA2 and PDX1 levels are also sufficient to rescue β-cell function in *βra*^*Het*^ islets, a similar experiment was performed to evaluate insulin secretion. Confirming our hypothesis and previous results, insulin secretion was impaired in *βra*^*Het*^ islets and rescued to control levels when FOXA2 was overexpressed similarly to PDX1 levels in Figure 8A (Figure 8B). To strength our conclusions, we assessed PDX1 levels by immunostaining in control, *βraKO,* and *βraKO* mice overexpressing a constitutively active form of S6K (*βraKO;caS6K)* previously described [8]. As previously published, PDX1 levels were decreased in β-cells with Raptor deletion [7,9]. Notably, genetic reconstitution of S6K activity increases PDX1 levels in β-cells with Raptor deletion (Figure 8C).

## 4 Discussion

The current studies provide novel insights into how mTORC1 regulates the adaptation of β-cell to HFD. We showed that deletion of one raptor allele is sufficient to impair glucose homeostasis by a defect in insulin secretion. The studies herein also identified that 1) heterozygous deletion of Raptor in β-cells renders these cells more susceptible to lipotoxicity and HFD conditions, 2) mTORC1 regulates β-cell genes that are critical for the adaptation to HFD such as *Ins1*, *MafA*, *Ucn3*, *Glut2* and *Glp1r*, 3) a novel AKT/FOXA2/PDX1 axis activation regulating β-cell adaptation to HFD in conditions of decrease in mTORC1 signaling (Figure 8D). The increase in FOXA2 levels rescuing PDX1 levels and insulin secretion in islets with heterozygous deletion of raptor suggest that this pathway could play a role in humans treated with mTORC1 inhibitors [33,34]. In addition, it has been hypothesized that in conditions that require adaptive cell proliferation, such as weight increase or metabolic syndrome, mTORC1 inhibitors could contribute to the development of new-onset diabetes after transplantation (NODAT) development [4,[35], [36], [37], [38]]. Therefore, studying the molecular interactions between mTORC1 signaling, FOXA2, and PDX1 and the downstream effects of this pathway in human islets from donors treated with mTORC1 inhibitors, would provide clinical insight, and guide the development of personalized approaches to optimize patient outcomes. 4) These studies demonstrate a pathophysiological role for mTORC1-dependent negative feedback loop in adaptation of β-cells to diet induced obesity.

Our group and others have shown that inhibition of mTORC1 in mouse genetic models and mTOR inhibitors augment apoptosis and/or impair β-cell function [[7], [8], [9],^11^,^12^,[39], [40], [41], [42], [43]]. However, raptor appears to be dispensable for β-cell development as mice with total deletion of raptor are born with normal β-cell mass [[7], [8], [9]]. It is not surprising that *βra*^*Het*^ exhibited no phenotype on β-cell growth and function in normal conditions. Therefore, assessments of fasted and fed insulin and glucose levels were normal in *βra*^*Het*^ mice in regular chow (Figure 1B–H). In contrast to mice with complete deletion of Raptor in β-cell (*βraKO)*, decreased Raptor levels were not sufficient to alter proinsulin content [8]. Although insulin content per cell was significantly decreased in *βra*^*Het*^ mice, static glucose-stimulated insulin secretion and intracellular calcium levels were similar to control mice (Figure. 2). In addition, islet morphology was also comparable to control mice (Figures. 1I-L), demonstrating that a decrease in raptor levels is not critical to maintaining adult β-cell mass and function. Together, our data demonstrate that partial Raptor deletion has no deleterious effects on β-cell mass and function in normal diet conditions. Furthermore, the decrease in raptor levels appears to have no impact on maintaining basal levels of pS6 (Figure 1, Figure 4). This finding is consistent with the absence of a phenotype in normal diet conditions. However, notable differences in pS6 levels emerge when mTORC1 activity is challenged with palmitate or amino acids after starvation and this explains in part the phenotype of these mice when exposed to HFD (Figure 4J and Supp Figure. 1).

mTORC1 activity is highly upregulated in the liver, fat, muscle and pancreatic islets of obese and high-fat-fed rodents and in islets of T2D humans [2,5,[44], [45], [46], [47]]. One consequence of chronic mTORC1 hyperactivation is the induction of an S6K1-dependent negative feedback loop leading to attenuation of AKT signaling in multiple tissues and insulin resistance [[2], [3], [4], [5]]. The current published evidence supports the concept that mTORC1 has a biphasic regulatory pattern that is consistent with the widely accepted model of β-cell deterioration ‘compensation/decompensation switch’ during the progression of T2D [15,[47], [48], [49]]. Metabolic stressors such as insulin resistance and nutrient excess increase β-cell mTORC1 in the initial functional compensatory phase [50]. This correlates with hyperinsulinemia and compensatory β-cell hypertrophy and hyperplasia, suggesting that mTORC1 is a key positive regulator of β-cell function and mass [50]. Consistent with this concept, previous studies have shown an improvement of the β-cell mass and function in states of insulin resistance with mTORC1 inhibitors [10,11]. Thus, it would have been anticipated that glucose homeostasis would have been improved in the *βra*^*Het*^ mice when fed HFD. Surprisingly, *βra*^*Het*^ mice exhibit defective adaptation to HFD with β-cell area reduction by increase in apoptosis suggesting that β-cells in these mice are more susceptible to metabolic changes induced by dietary fat. This was validated by increase susceptibility of *βra*^*Het*^ β-cells to lipotoxicity. The increase susceptibility to lipotoxicity and impaired mTORC1 activity upon palmitate treatment is consistent with previous studies showing that glucose and palmitate induce activation of mTORC1 and ER protein load in β-cell [[51], [52], [53]]. Together, these results highlight the importance of adequate levels of Raptor for the function and survival of the β-cell in conditions of lipid excess and during insulin resistance induced by HFD. The reduction in adaptive responses during HFD associated with reduction in critical β-cell genes for β-cell identity such as *Ins1*, *MafA*, *Ucn3*, *Glut2*, *Glp1r* and more importantly, *Pdx1* [7,54,55] is consistent with recently published results showing that complete deletion of Raptor alters function and maturation of the β-cell by controlling *Pdx1*, *Mafa*, and *Ucn3* among others [7,9]. Further, published and current studies are consistent with a model in which complete Raptor deletion results in low Ucn3 and Glut2 followed by a defect in β-cell identity [7]. However, the conditions of complete mTORC1 inhibition are not observed in physiology or during disease states. Therefore, the current work extends previous published studies by showing that mTORC1 activity is required for β-cell adaptation in a model of T2D by maintaining the activity of the mTORC1-mediated feedback inhibitory loop (Figure. 6). This is consistent with the concept that mTORC1 activation could initially play a physiological role in adaptation nutrient excess and obesity. However, chronic mTORC1 hyperactivation caused by sustained nutrient overload induces an mTORC1/S6K1-dependent negative feedback loop causing β-cell exhaustion, functional collapse and ultimate cell death (decompensatory phase) [[56], [57], [58], [59]]. Finally, the resistance of *βra*^*Het*^ β-cells to thapsigargin induced apoptosis is intriguing and likely consistent with previously published experiments showing that inhibition of mTORC1 has a protective effect alleviating ER stress possibly by decrease in protein synthesis [10,11,60]. Taken together, these studies suggest that decrease mTORC1 activity can regulate β-cells survival to specific stressors.

Previous work by our group and others have shown that PDX1 levels are regulated by mTOR signaling [7,9,40,42,61]. However, how mTOR regulates PDX1 has not been directly explored. Mice with β-cell-specific deletion of Raptor exhibit a reduction in *Pdx1* mRNA expression [9]. Using heterozygous deletion of raptor in β-cells, we show normal PDX1 levels when fed regular chow (Sup. Figure. 3). In HFD, PDX1 mRNA levels increase in controls but this increase is limited in *βra*^*Het*^ mice (Figure. 6A). This limited increase in PDX1 levels was confirmed at the protein levels by WB and staining (Figure 6D–E). Recently, our group identified a novel mTORC1/FOXA2 axis as a link between mTORC1 and transcriptional regulation of key genes responsible for α-cell function and survival [30]. In β-cells, FOXA2 has been previously proposed to regulate PDX1 levels [27] and low PDX1 levels have been associated with dedifferentiation and impaired insulin secretion [25,27,62]. Given the key role of FOXA2 in β-cell regulating maturation, PDX1 expression and insulin secretion, we considered FOXA2 as a potential candidate to regulate PDX1 in HFD conditions in *βra*^*Het*^ mice [25,27,[63], [64], [65]]. We found that although there were no differences in the total levels of FOXA2, the levels of phosphorylated FOXA2 were significantly higher and this resulted in retention of FOXA2 in the cytoplasm of β*-*cells from the *βra*^*Het*^ mice in HFD compared to controls (Figure 7B–C). The mechanistic role of FOXA2 in the defects observed in *βra*^*Het*^ mice in HFD was further confirmed by rescuing PDX1 levels and insulin secretion. Together, these data suggest that in conditions of reduced mTORC1, the AKT/FOXA2 axis activation plays a critical role in regulating PDX1 levels, a key transcription factor necessary for β-cell adaptation to HFD.

## 5 Conclusion

In summary, our results uncover that Raptor levels are critical in β-cells adaptation to insulin resistance but not in normal conditions and demonstrate the importance of the negative feedback inhibition of mTORC1/S6K on IRS/AKT signaling. Deletion of only one allele of raptor is sufficient to impair insulin secretion when fed a HFD, increases apoptosis without altering proliferation rate, and this is accompanied by abnormalities in transcription of critical β-cell genes including *Pdx1*. These findings also support the concept that a decrease in the mTORC1/S6K negative feedback loop leads to an increase in AKT activity and FOXA2 phosphorylation/cytoplasmic retention leading to reduced levels of PDX1. The importance of this AKT/FOXA2/PDX1 axis in adaptation of β-cells to insulin resistance is only observed in conditions of reduced mTORC1 activity. The regulation of this axis could have implications in humans treated with mTOR inhibitors. Finally, these studies also reveal that increasing FOXA2 levels in islets with reduced mTORC1 activity rescue PDX1 levels and insulin secretion, highlighting the importance of the AKT/FOXA2/PDX1 axis as a possible therapeutic tool to improve β-cells in conditions of insulin resistance.

## Footnotes

Author contributions. M.B.-R., R.A.L, J.P·W.-D.-C., C.L. and J.A. performed the experiments and analyzed results. M.B.-R. and E.B.-M. wrote the article and designed the experiments. M.A.R and M.N.H. generated mice. M.B.-R., G.L. and E.B.-M. contributed to discussion and reviewed and edited the manuscript. M.B.-R. and E.B.-M. are the guarantors of this work and, as such, had full access to all the data in the study and takes responsibility for the integrity of the data and the accuracy of the data analysis.

## Declaration of Competing Interest

The authors declare that they have no known competing financial interests or personal relationships that could have appeared to influence the work reported in this paper.

## Data Availability

Data will be made available on request.
