# Supplementary material for: Raptor levels are critical for β-cell adaptation to a high-fat diet in male mice
Source: Mol Metab. 2023 Jul 7;75:101769. doi: 10.1016/j.molmet.2023.101769 (PMC10391668; doi:10.1016/j.molmet.2023.101769)

Supplemental Figure 1

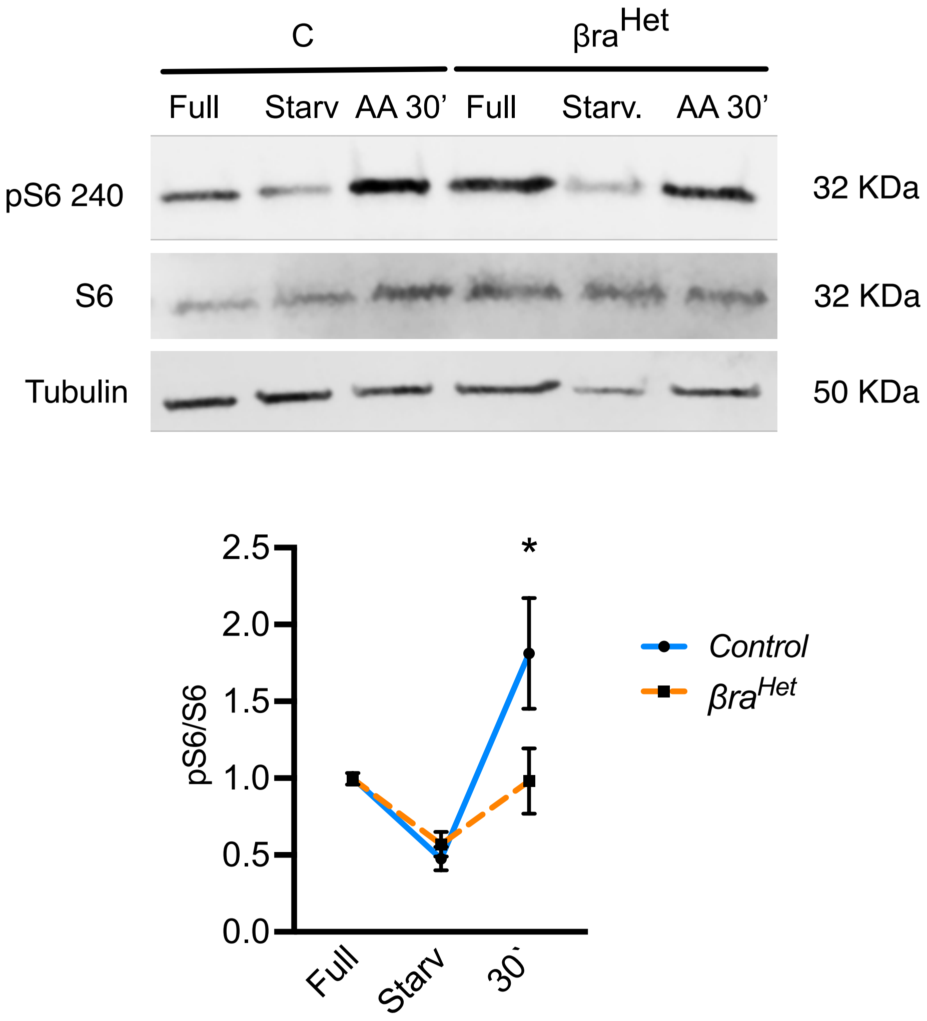

Supplemental Figure 2

a

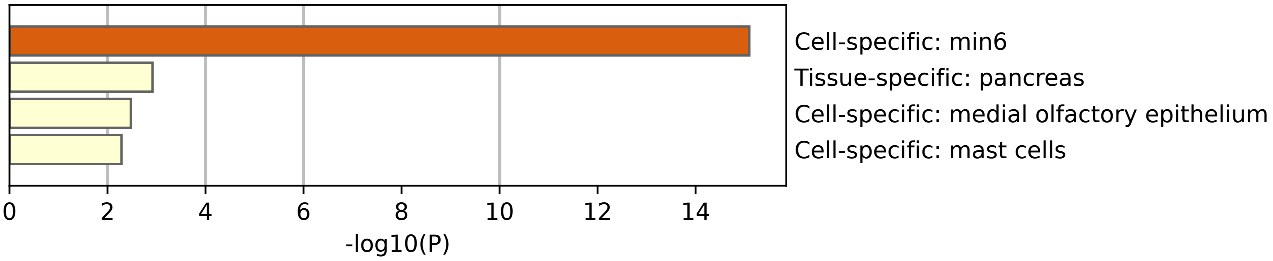

b

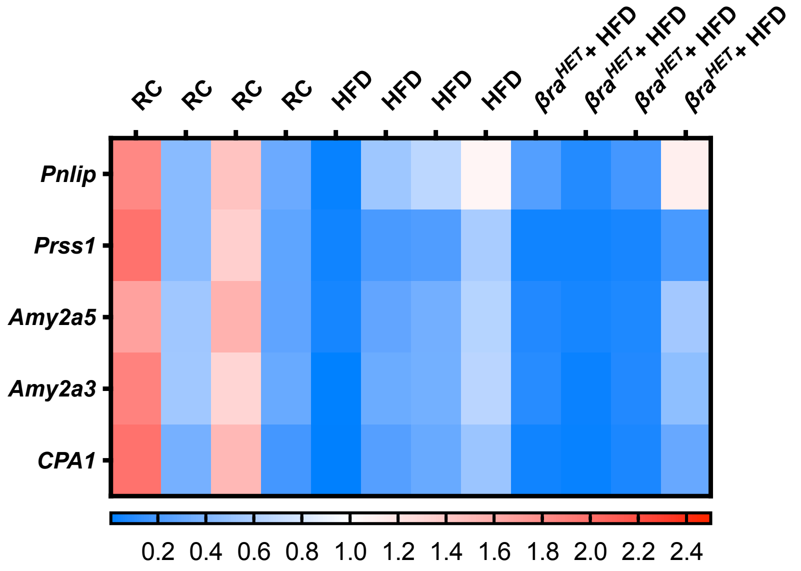

c

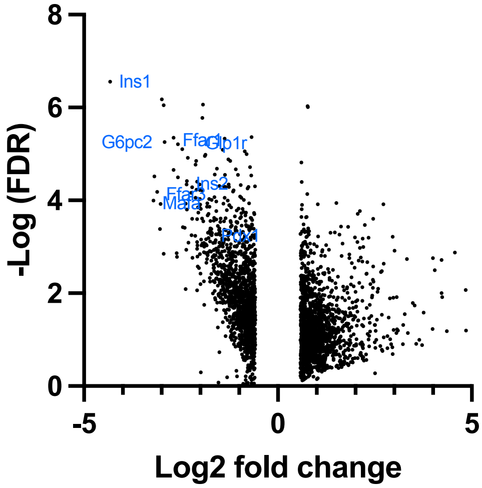

Supplemental Figure 3

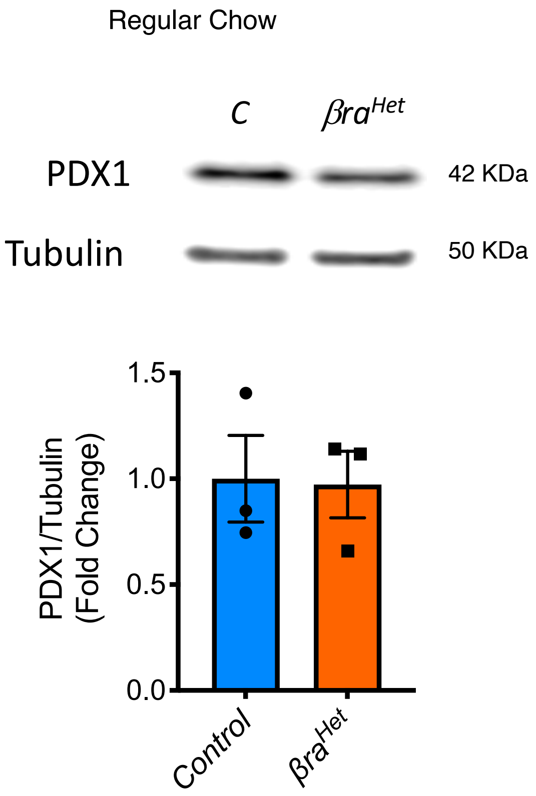

Supplemental Figure 4

a

Pdx1_targets.mouse

# TF	Target	Type	Reference
Pdx1	Fam3b	Activation	18708173
Pdx1	Gcg	Unknown	11825903
Pdx1	Ins2	Activation	7568086
Pdx1	Ins2	Unknown	10426567;10567373;20886630
Pdx1	Neurog3	Activation	19487809
Pdx1	Nr5a2	Activation	12972592
Pdx1	Pax4	Unknown	11825903
Pdx1	Slc2a2	Unknown	19651901
Pdx1	St18	Unknown	23236509
Pdx1	Tshz1	Unknown	25918232

b

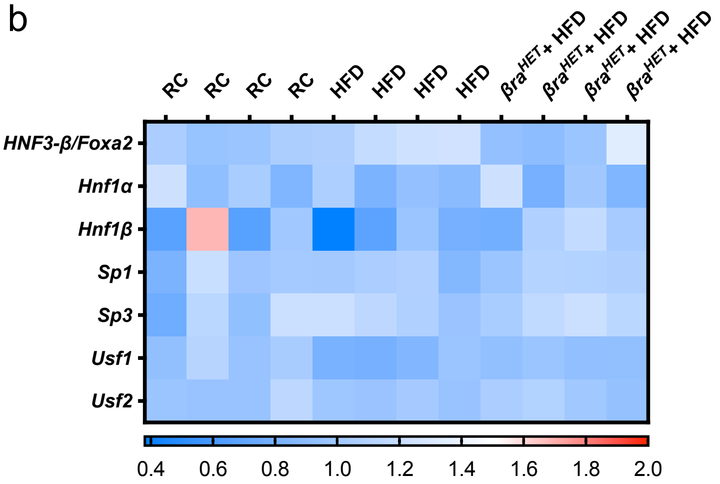

c

TF	# of overlapped target genes	P value	FDR
Foxa2	3	6.82e-07	3.57e-06
Onecut1	2	4.87e-06	1.70e-05
Mafa	2	6.26e-06	2.10e-05
Neurod1	2	1.15e-05	3.46e-05
Mitf	2	8.57e-05	1.67e-04
Foxo1	2	2.55e-04	3.95e-04

d

Pdx1_regulators.mouse

# [TF]	[Target]	[Type]	[Reference]
Atf3	Pdx1	Repression	21821004
Egr1	Pdx1	Activation	17150967
Foxa1	Pdx1	Unknown	19141476
Foxa2	Pdx1	Activation	12145169;9315659
Foxa2	Pdx1	Unknown	10652343;12052878;19141476
Foxo1	Pdx1	Repression	12488434;21335550
Foxo1	Pdx1	Unknown	16644672
Maf	Pdx1	Activation	12551916
Mafa	Pdx1	Activation	12551916;17149590
Mafa	Pdx1	Unknown	18522939
Mlxipl	Pdx1	Repression	20934404
Neurod1	Pdx1	Unknown	20448145
Nkx2-2	Pdx1	Activation	16147997
Onecut1	Pdx1	Activation	12781686
Pax4	Pdx1	Activation	14729487
Pax6	Pdx1	Unknown	12052878
Pparg	Pdx1	Unknown	18718916
Sox9	Pdx1	Activation	21829703
Sox9	Pdx1	Unknown	20448145
Usf1	Pdx1	Activation	8567692

Supplemental Figure 5

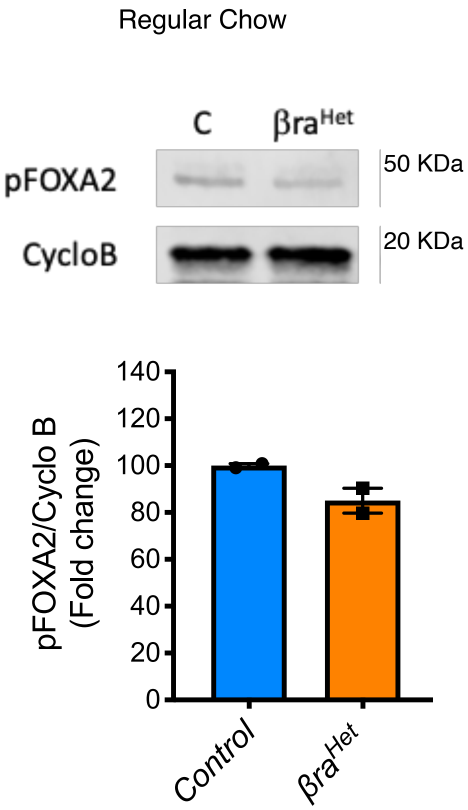

Supplemental Figure 6

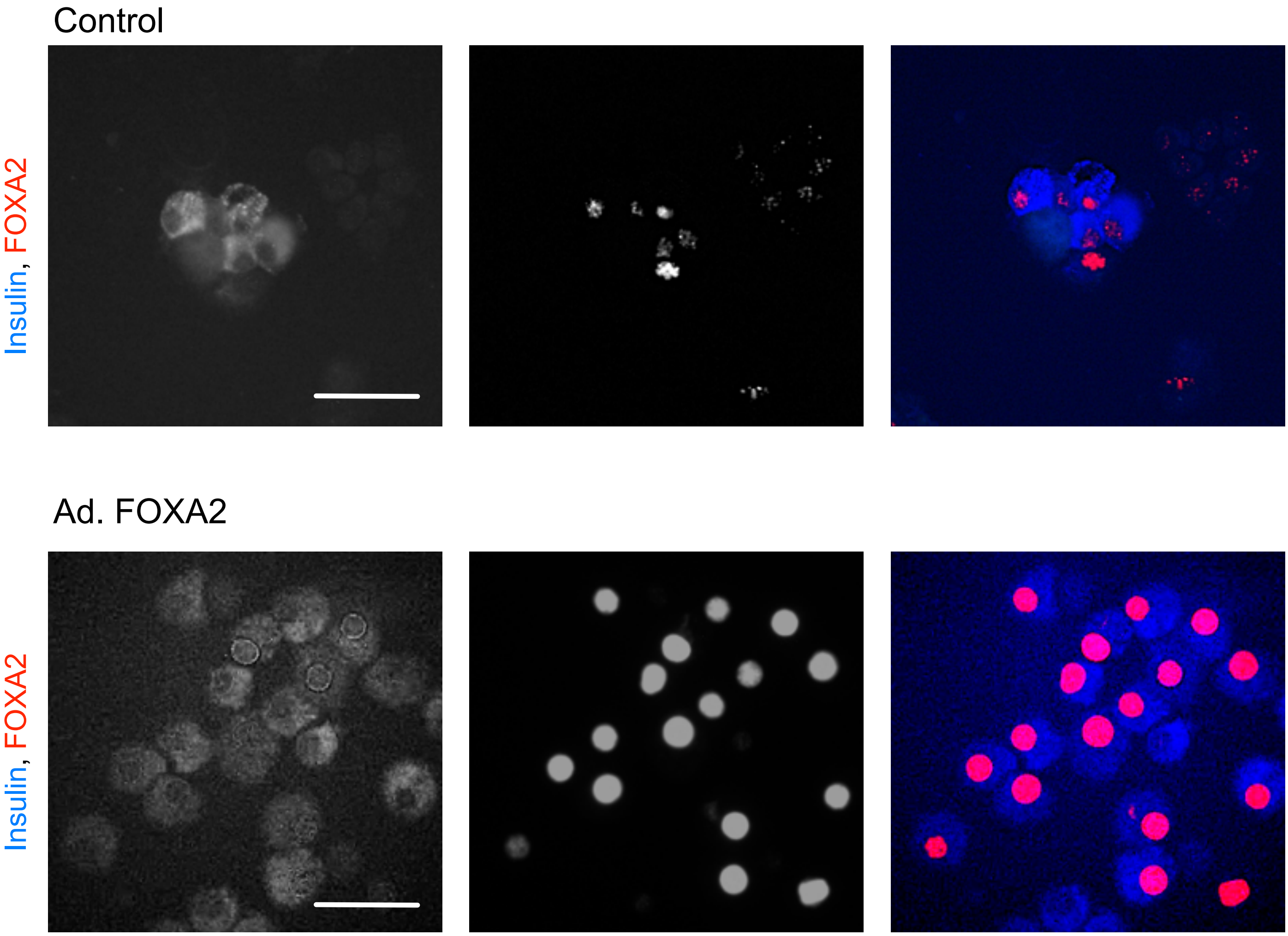

Supplement: Multimedia component 1 — . [file mmc1.pdf]
