# Supplementary material for: Raptor levels are critical for β-cell adaptation to a high-fat diet in male mice
Source: Mol Metab. 2023 Jul 7;75:101769. doi: 10.1016/j.molmet.2023.101769 (PMC10391668; doi:10.1016/j.molmet.2023.101769)
Supplement: Multimedia component 2 [file mmc2.docx]

**Supplemental Table 1. Antibodies**

| **Antibody** | **Specie** | **Source** | **Catalog** | **Concentration** |
| --- | --- | --- | --- | --- |
| Active Caspase 3 BV650 | Rabbit | BD Trans | 564096 | 5 ul/test |
| CycloB | Rabbit | Thermo Fisher | PA1027A | 1:5000 |
| FOXA2 | Mouse | Abcam | 60721 | 1:500 |
| Insulin | Guinea Pig | Dako | A0564 | 1:400 |
| Insulin APC Conjugated | Rat | R&D Systems | IC1417A | 1 ul/test |
| Ki67 | Rabbit | Vector | NCL-Ki67p/VPK451 | 1:200 |
| pAKT (308) | Rabbit | Cell Signaling | 4056 | 1:1000 |
| PDX1 | Guinea Pig | Abcam | Ab47308 | 1:1000 |
| pFOXA2 (156) | Rabbit | Thermo Scientific | 710680 | 1:500 |
| Proinsulin | Mouse | R&D Systems | MAB13361 | 1:400 |
| pS6 (240) | Rabbit | Cell Signaling | 5364 | 1:2000 |
| Raptor | Rabbit | Cell Signaling | 2280 | 1:1000 |
| S6 | Mouse | Cell Signaling | 2317 | 1:1000 |
| Tubulin | Mouse | Sigma | T5168 | 1:3000 |
